# Management of Early Post-Operative Complications of Esophageal Atresia With Tracheoesophageal Fistula: A Retrospective Study

**DOI:** 10.7759/cureus.11904

**Published:** 2020-12-04

**Authors:** Muhammad Khalid Syed, Ahmad A Al Faqeeh, Alsayed Othman, Talal Almas, Tarek Khedro, Reema Alsufyani, Dana Almubarak, Rehab Al Faqeh, Saifullah Syed, Sabahat K Syed

**Affiliations:** 1 Consultant Pediatric Surgery, King Fahad Hospital, Al Bahah, SAU; 2 Pediatric Surgery, King Fahad Hospital, Al Bahah, SAU; 3 Pediatric Surgery, Alazhar University, Assiut, EGY; 4 Internal Medicine, Royal College of Surgeons in Ireland, Dublin, IRL; 5 Internal Medicine, Jinnah Sindh Medical University, Karachi, PAK

**Keywords:** esophageal atresia, tracheoesophageal fistula

## Abstract

Background

Esophageal atresia (EA) with tracheoesophageal fistula (TEF) is a rare congenital malformation of the trachea and the esophagus. While the condition can result in a debilitating clinical picture, its outcomes have significantly ameliorated in recent times. The diminishing mortality associated with the disease can be attributed to a myriad of factors, including surgical advances, specialized anesthetic care, and categorical ventilator provision. These advances have resulted in increased survival rates even in premature infants who present with exceedingly low birth weights. Nevertheless, the mortality surrounding the condition still remains exceedingly high in some parts of the world, including the Middle East and Asia. The aim of the present study is to identify and outline the management of the postoperative complications that are intricately linked with soaring mortality rates.

Methods

We conducted a single-center retrospective study, three years in duration, of all the patients who were operated for esophageal atresia with tracheoesophageal fistula. The exclusion criteria included patients who died before the operation and those who were referred to other centers for management. The study evaluated several factors, including the various postoperative complications, their adept management, and the eventual outcomes. Data pertaining to the patient demographics, treatment, and radiological and laboratory findings was obtained and eventually analyzed using the Statistical Package for Social Sciences (SPSS) version 23.0 (IBM Corp., Armonk, NY, USA) software.

Results

The present study included a total of 12 cases diagnosed in our hospital during the aforementioned study period. Of these patients, two patients (16.7%) died before operation because of associated severe congenital anomalies such as cardiac pathologies. Three patients were referred to other centers for management. These patients were excluded from our analysis. The remaining seven patients were included in our analysis. In our study, gastroesophageal reflux was the most common postoperative complication and was noted in six patients. Leakage of anastomosis was noted in two patients. Lung collapse was noted in merely one patient and was thus the least common complication. The overall mortality rate hovered around 28.6%.

Conclusions

While most patients who are surgically managed for esophageal atresia with tracheoesophageal fistula develop postoperative complications, these complications are amenable to conservative management through the means of antibiotics, ventilator support, and total parenteral nutrition.

## Introduction

Esophageal atresia (EA) with tracheoesophageal fistula (TEF) is a congenital anomaly that occurs due to an atretic esophagus and an aberrant anatomical relationship between the trachea and the esophagus [1]. EA with TEF occurs due to a failure of the laryngotracheal tube to separate into esophagus and trachea during the gestational period [2]. While the condition is broadly divided into five types (type A through type E), type C, which encompasses EA with distal TEF, is noted to be the most prevalent and is observed in 86% of the cases [1-3]. Notably, the overall incidence of EA with TEF hovers around one in every 10,000 live births [2]. Prenatally, the diagnosis of EA with TEF can be suspected if indications of polyhydramnios, such as maternal dyspnea, persist. In such instances, further radiological workup through the means of a routine antenatal ultrasound scan divulges an abnormally small stomach, which further prompts the diagnosis of the condition [4]. However, due to its low sensitivity and specificity, ultrasound imaging is not considered a definitive diagnostic modality [1,3]. Current medical literature suggests that fetal magnetic resonance imaging (MRI), although employed with caution, is an efficacious modality in confirming suspicions of the condition [4]. Clinically, EA with TEF presents with signs of respiratory distress, such as grunting, subcostal recession, and nasal flaring, coughing, and pneumonia [5]. In cases that are not appropriately diagnosed prenatally, the presence of these clinical symptoms warrants further diagnostic workup. The gold standard diagnostic modality is the failure to pass a nasogastric tube beyond the initial 10 centimetres [4,5]. Radiograph imaging of the abdomen often reveals the presence of gas, thereby unmasking the presence of distal TEF. In the same vein, the absence of gas in the abdomen might indicate the presence of an isolated EA [1]. Furthermore, the concurrent presence of other vertebral defects, anal atresia, cardiac defects, tracheo-esophageal fistula, renal anomalies, and limb abnormalities (VACTERL defects), also substantiates the diagnosis. The preoperative management draws upon feeding gastrostomy, intravenous fluid administration, and low suction within the upper esophageal pouch in order to avoid the risk of aspiration. Due to advanced surgical care, the prognosis has significantly ameliorated [6]. The most common procedure used to rectify the EA with TEF is a right thoracotomy, which encompasses the separation of the abnormal communication between trachea and the esophagus and a subsequent primary end-to-end anastomosis of the esophagus [3,7]. Nevertheless, postoperative complications such as anastomotic leakage remain a major challenge for surgeons due to their occurrence despite the advents in modern surgical care. The most commonly occurring complications are anastomosis leakage, tension pneumothorax, and sepsis [7,8]. Moreover, the long term complications of the surgery include esophageal strictures, reoccurrence of TEF, tracheomalacia, gastroesophageal reflux, dysphagia, recurrent pneumonia, and cough. The aim of our study is to assess both the incidence and management of the early postoperative complications of EA with TEF in our hospital.

## Materials and methods

A retrospective cross-sectional study was conducted in the Pediatric Surgical Unit at King Fahad Hospital, Al Bahah, Saudi Arabia. Patients with a confirmed diagnosis of EA with TEF who were operated during the study period, from 2017 to 2020, were included in the present study. Patients who died before being operated and those that were referred to other centers upon their parents’ volition were excluded from the analysis. Data regarding the patients’ history, clinical examination findings, postoperative complications, and outcomes were recorded. The data were primarily collected by perusing the patients’ computed records and files. Due ethical approval was obtained from the ethical review board at the institution. The data was analyzed using the Statistical Package for the Social Sciences (SPSS) 23.0 (IBM Corp., Armonk, NY, USA) software and was then tabulated across the various parameters studied. 

## Results

**Figure 1:**
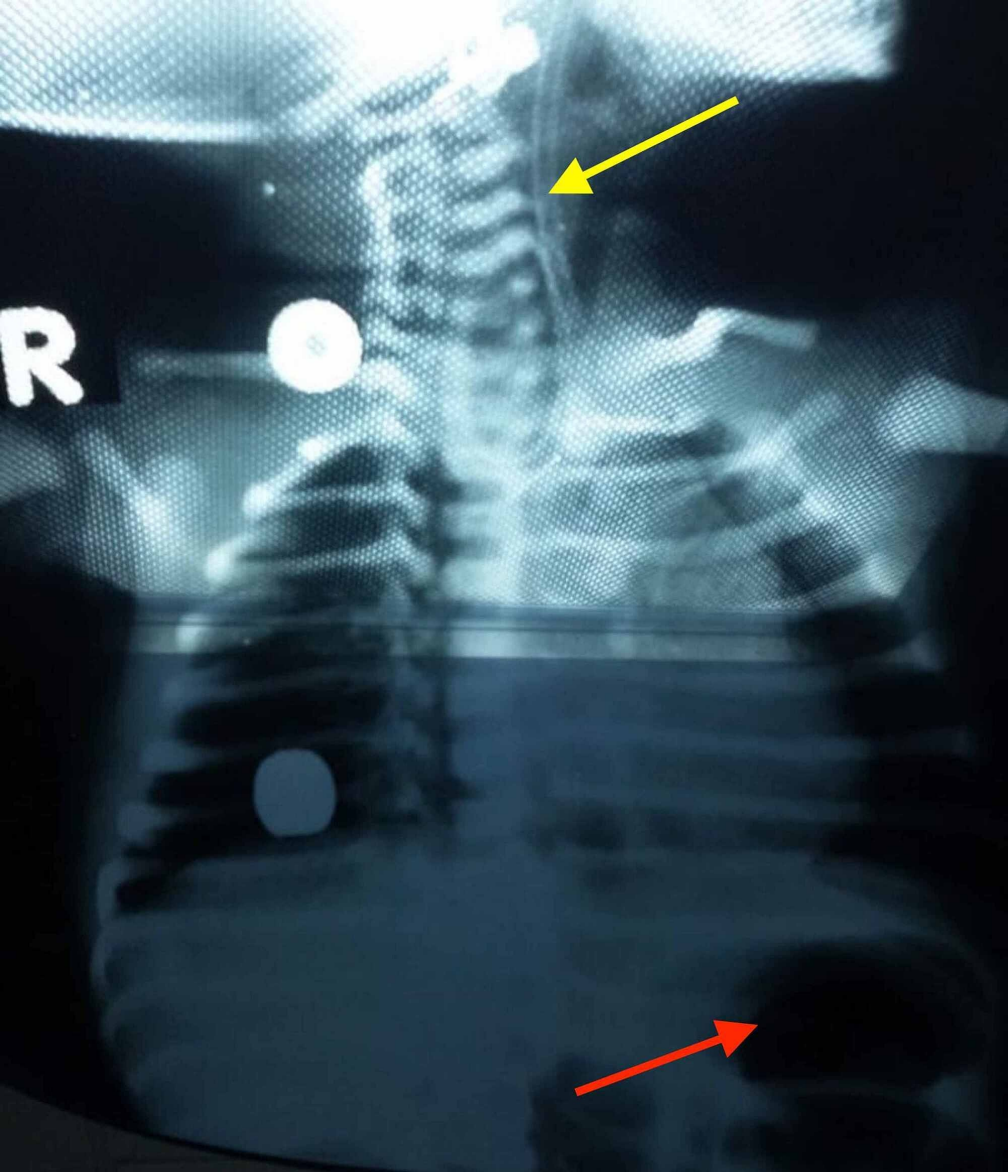
A preoperative plain radiograph demonstrating coiling of the NG tube in the upper pouch of the esophagus (yellow arrow) and gas in the stomach (red arrow), indicating a tracheoesophageal fistula. NG: nasogastric

A total of 12 patients with a confirmed diagnosis of EA with TEF were considered in the present study. Of these, five were excluded due to subsequent referral to other centers, leaving a total of seven patients that were studied for complications in the current study. Considering the varying clinical pictures that EA with TEF can present with, the time of diagnosis was deemed a noteworthy indicator of prognosis. While 83% of the patients were diagnosed within 24 hours of birth, approximately 17% of the patients were diagnosed more than 24 hours of birth. As stated previously, postnatal parameters, such as prematurity and a low birth weight, are associated with adverse postoperative outcomes. Therefore, it was deemed apt to analyze the postnatal status of the study participants. Of the seven patients, four were premature and six presented with a low birth weight. The clinical presentations included excessive salivation and frothing, inability to feed, failure to pass a nasogastric tube, and respiratory distress. In all of our cases, radiograph imaging revealed coiling of the nasogastric tube in the upper esophageal pouch and gas within the gastrointestinal tract, indicating a tracheoesophageal fistula (Figure 1). 

**Figure 2:**
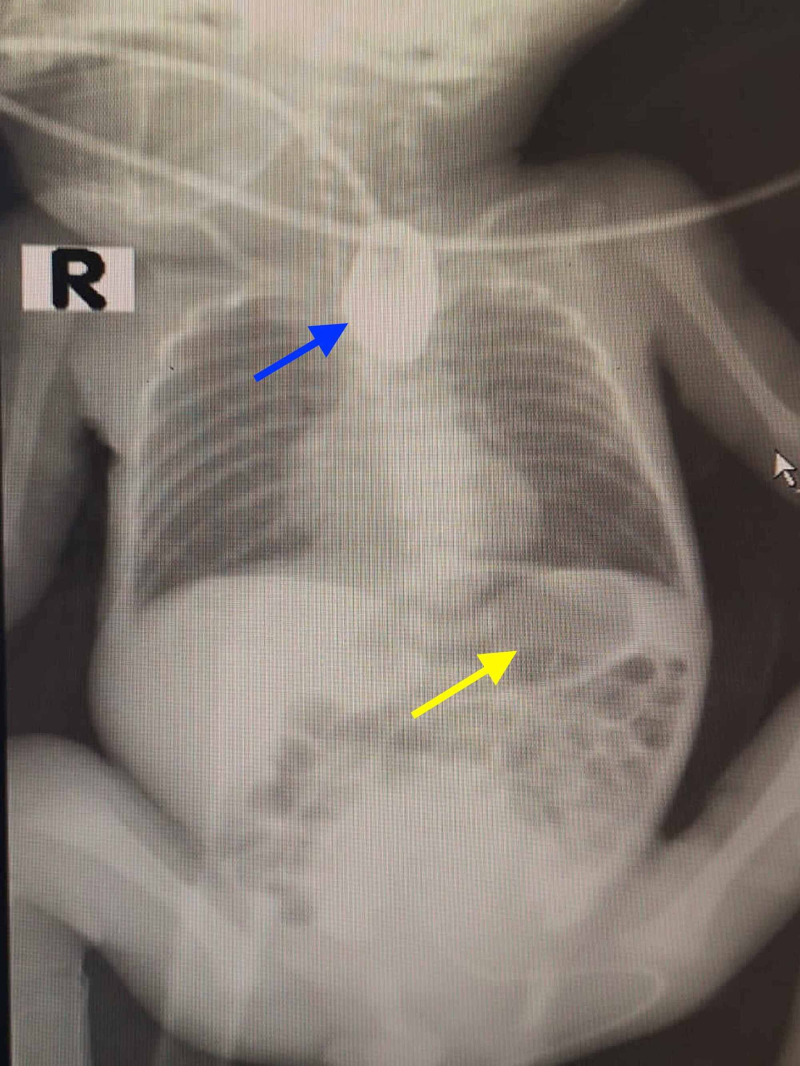
A preoperative radiograph showing the presence of contrast material within the upper esophageal pouch (blue arrow). The presence of gas in the stomach is also noted (yellow arrow).

Furthermore, the preoperative administration of contrast material in the upper esophageal pouch was noted to be confined only to the upper esophageal pouch and failed to progress caudally due to the atretic esophagus (Figure 2). 

Preoperatively, all patients were on mechanical ventilation, and slow suction of the upper esophageal pouch was performed in order to curb the risk of aspiration. Notably, all patients presented with a type C esophageal atresia. Right posterolateral thoracotomy, ligation and division of the distal tracheoesophageal fistula, and primary end-to-end anastomosis of both ends of the esophagus were performed in all patients. The spectrum of the various early postoperative complications is elucidated in Table 1 below.

**Table 1:** The varied spectrum of the early postoperative complications observed within our study population.

Complication	Frequency (n)	Recovered from complication	Died from complication	On follow-up
Gastroesophageal reflux	6	3	1	2
Septicemia	2	1	1	0
Leakage of anastomosis	2	2	0	0
Lung collapse	1	1	0	0

**Figure 3:**
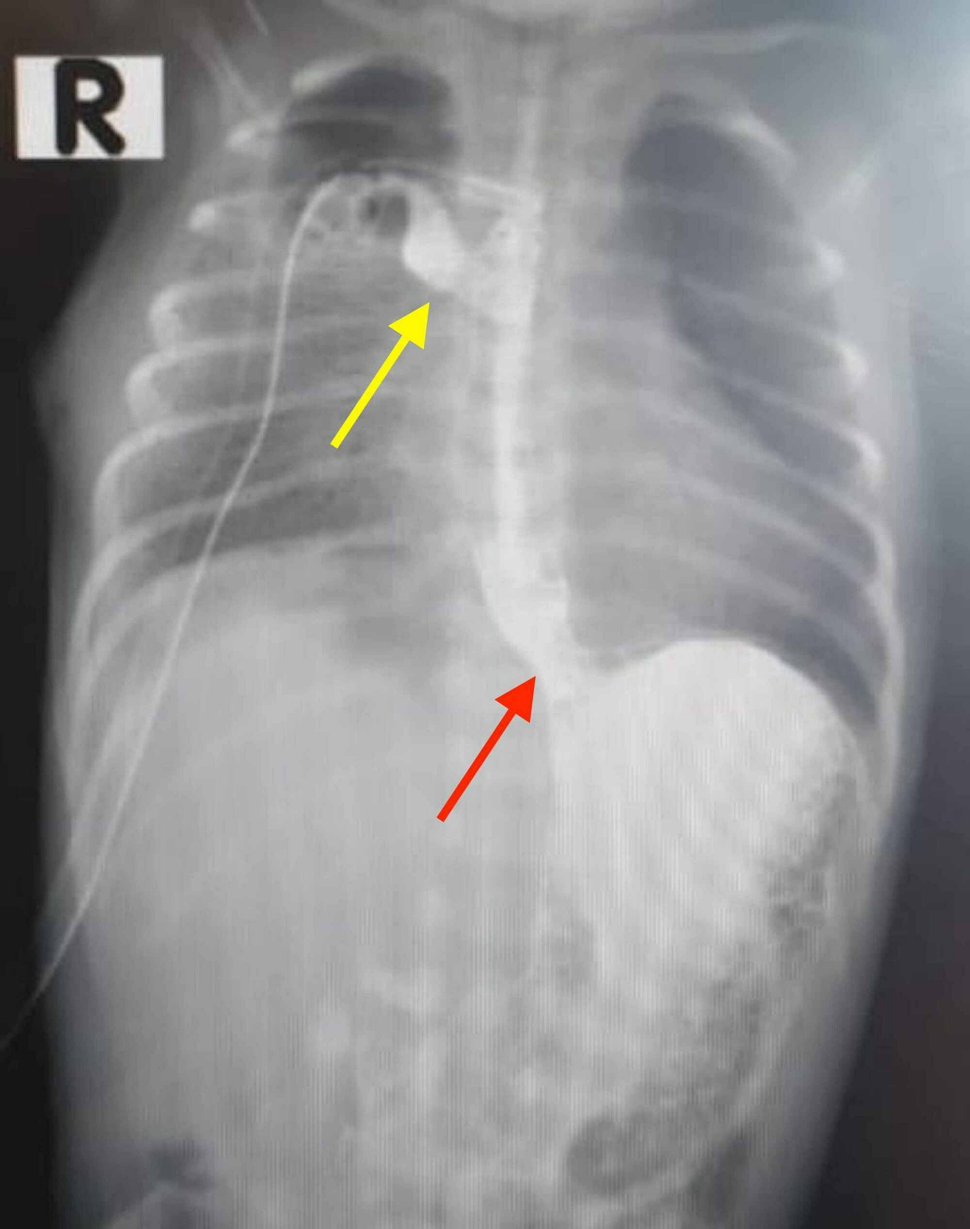
A postoperative radiograph showing minor leakage of the contrast from the anastomosis after the surgical repair of EA with TEF (yellow arrow). Gastroesophageal reflux is also noted (red arrow). EA with TEF: esophageal atresia with tracheoesophageal fistula.

Within our study population, the commonest postoperative complication was gastroesophageal reflux, which was noted in six patients and is delineated by Figure 3

Gastroesophageal reflux was managed conservatively by head-up positioning, lateral positioning, anti-reflux formula milk, pro-kinetic medication and proton pump inhibitors. While the majority of the patients demonstrated good results, one patient died at the age of four months with aspiration pneumonia due to severe gastroesophageal reflux. A further two patients developed severe septicemia. Imperatively, two patients were noted to present with leakage of the curated anastomosis. In one of these two patients, the leakage was minor and was diagnosed by oral water-soluble contrast in the presence of a nasogastric tube. This minor leakage was managed conservatively by delaying the commencement of feeding and prolonged total parenteral nutrition. This resulted in a gradual cessation of the patient’s anastomotic leak. The second patient presented with a major leakage. In order to combat the major leak, irrigation of the thoracic cavity by saline through a chest tube, along with all the procedures typically employed for minor leaks, was performed. To our knowledge, management through irrigation of the thoracic cavity has not been reported in the literature. Lung collapse was noted merely in one patient, who improved after one week of ventilator support, prophylactic antibiotic therapy, and chest physiotherapy.

## Discussion

Until 1941, when the first successful repair of EA with TEF was performed, most patients afflicted with this congenital anomaly died within days or even hours of surgery. Over time, as the surgical management of the condition evolved, what once was a diagnosis of imminent death became one in which survival is the expected outcome. In the past few decades, mortality due to the condition has been significantly reduced [9]. However, complications and morbidities are still a major issue, often due to factors such as associated chromosomal abnormalities, varying degrees of VACTERL defects, timeliness of diagnosis, and especially the limited capabilities of the healthcare infrastructure in some parts of Middle East and Asia [9,10].

Out of the seven patients in this paper, two died. Of these, one died due to septicemia on the 10th day of life and the other due to aspiration pneumonia secondary to severe gastroesophageal reflux at the age of four months. In the literature, gastroesophageal reflux is reported to occur in 30-82% of surgical repairs, and 30% of these patients end up requiring further anti-reflux operations [10]. All five of our patients who survived the operation developed gastroesophageal reflux, three of whom improved exclusively through conservative management. The other two patients are currently being monitored closely. If their reflux status does not improve, they may require surgical intervention, with Nissen fundoplication being the first-line strategy [11]. Although gastroesophageal reflux does resolve spontaneously in most cases, it sometimes requires fundoplication within a few years of its initial onset [11,12]. Furthermore, two patients in the study developed a leakage of an anastomosis, one on day six (major leak) and the other on day 10 (minor leak). However, both leaks sealed spontaneously after conservative management through a nil per os approach, intravenous fluids, antibiotic therapy, and total parenteral nutrition [12]. In our study, the patient with the major leak experienced complications due to the bile in the chest tube, likely a direct result of reflux [12]. This necessitated irrigation of the right thoracic cavity using saline, which provided a buffer against the irritating bile and gastric fluid present in their thorax [12-14]. This irrigation further helped in healing the leak by preventing adhesion and empyema formation [15]. To our knowledge, this approach has not yet been reported in the literature. In some of these cases, operative measures are required if the leak does not resolve [15]. Of note, another one of our patients developed right lung collapse but then improved spontaneously. The reason for this collapse is still unclear; however, we suspect it to have been due to a mucus plug in the right main bronchus.

While our study highlights some of the key short-term complications that ensue in the aftermath of surgically managing EA with TEF, the congenital malformation can also lead to a myriad of other long-term complications that are not evidenced by this study [15-18]. One such complication is an anastomotic stricture, the most common complication that results from the postoperative repair of EA [16,17]. These very often result in esophageal stenosis and strictures, and in comparing the surgical interventions for EA repair, stenosis was more common after thoracoscopic repairs versus thoracotomies [13-18]. In 2016, one large retrospective study of 430 patients in India reported the survival at 29.77% and mortality at 68.84% [15]. Data from other institutions in the 1990s shows survival rates to be higher, between 85-90% [16-18]. This marked difference in high-volume tertiary centers in India, despite being 20 years later, is due to a multitude of factors, including delayed diagnoses (>50% of neonates diagnosed more than 24 hours after birth had developed pneumonia), timely transportation to a pediatric surgical facility, and the availability of resources such as beds, neonatal ventilators, and trained neonatal intensive care unit staff [15-18].

In this study, the mortality rate of patients who underwent operation was 28.5%. Despite it being a small study, this paper highlights the common important early short-term complications that are associated with EA with TEF as well as the successful management of these complications. Close follow-up is necessary in order to circumvent any further complications that may arise, especially in a more fragile neonatal population. 

## Conclusions

EA with TEF is a congenital malformation that can elicit a myriad of clinical ramifications. Due to the grave complications that are frequently associated with the condition, the condition should be managed by experienced pediatric surgeons in specialized centers. To combat a major anastomotic leakage, irrigation by saline through a chest tube is imperative in ameliorating the eventual disease outcomes.
